# Consumption of Dietary *Premna microphylla* Turcz Leaf Alleviates Functional Constipation via Regulating Gut Microbiota and Aquaporins Transport System in Rats

**DOI:** 10.3390/foods14203535

**Published:** 2025-10-17

**Authors:** Nan Wang, Mengxue Zhang, Li Zhang, Daoyuan Ren, Yan Zhao, Xingbin Yang

**Affiliations:** 1 Key Laboratory of Ministry of Education for Medicinal Resource and Natural Pharmaceutical Chemistry, College of Life Sciences, Shaanxi Normal University, Xi’an 710119, China; wangnan_vip@yeah.net (N.W.); nikoafk5323@gmail.com (M.Z.); 18292606003@163.com (L.Z.); yanzhao@snnu.edu.cn (Y.Z.); 2 Shaanxi Engineering Laboratory for Food Green Processing and Safety Control, Shaanxi Key Laboratory for Hazard Factors Assessment in Processing and Storage of Agricultural Products, College of Food Engineering and Nutritional Science, Shaanxi Normal University, Xi’an 710119, China; dyren@snnu.edu.cn

**Keywords:** *Premna microphylla* Turcz leaf, constipation, gut microbiota, aquaporin proteins, neurotransmitters

## Abstract

This study, for the first time, explored the preventive effects of a novel pectic polysaccharide from *Premna microphylla* Turcz leaves (PMTL) on experimental constipation. Diphenoxylate-induced constipation model (CM) rats were fed a standard rodent chow supplemented with or without PMTL (5%, 10%, and 20%) for 6 weeks. Supplementation of PMTL was shown to accelerate intestinal peristalsis, increase fecal water content, improve intestinal morphology, and suppress gut inflammation by facilitating the secretion of excitatory neurotransmitters (MTL, ACH, and SP) and decreasing the secretion of inhibitory neurotransexcitatory neurotransmittersmitters (SS and NO) in the CM rats. PMTL also reduced the expressions of the colonic aquaporins AQP3 and AQP4 in the CM rats to normalize the colonic water transport system. 16S rRNA gene sequencing revealed that PMTL relieved the gut microbiota disorder of the CM rats and promoted the proliferation of several beneficial bacteria, resulting in an increase in fecal short-chain fatty acids. These findings demonstrate that dietary PMTL consumption can ameliorate the development of constipation and PMTL can be considered as a great promising dietary supplement for alleviating constipation.

## 1. Introduction

As modern diets and lifestyles evolve, constipation has increasingly become a prevalent gastrointestinal complaint with significant implications for daily living and well-being [1]. Constipation can occur for many reasons, including inadequate dietary fiber or fluid, stress, physical inactivity, and depression [2,3]. Current studies have reported that constipation is closely related to intestinal microbiota, and their changes may impact the formation of feces and the content of short-chain fatty acids (SCFAs) [4,5,6]. Many studies show that gut microbiota produces SCFAs (e.g., acetic acid, propionic acid, and butyric acid) which not only directly affect the intestinal smooth muscle contraction but also adjust gut pH to promote intestinal peristalsis [6]. In addition, long-term constipation will also affect the integrity of gastrointestinal tract and the expression of water channel proteins, as well as abnormality of the enteric nervous system [7,8,9,10,11]. Traditionally, some drugs such as osmotic or secretory laxatives and dilatants are used to relieve constipation, but these therapies often cause side effects, such as colon nervous system damage and cardiovascular diseases [12,13,14]. In this regard, it is a trend and necessity to seek natural and friendly phytochemical agents to relieve constipation symptoms [12,15].

*Premna microphylla* Turcz leaves (PMTL), widely distributed in central and southern areas of China, are the classic raw materials for processing delicious food of traditional green tofu, which is known locally as “Shenxian” or “Guanyin” tofu for a long history in China [16,17]. PMTL is cultivated on a large scale as a commercially significant crop due to its multifarious applications and nutritional benefits [18,19]. It contains abundant pectins, crude fiber, carbohydrates, plant proteins, amino acids, minerals, and polyphenols [20,21,22]. Although the potential presence of antinutritional factors—such as phytates, tannins, or oxalates, which may interfere with nutrient absorption—was considered, available phytochemical analyses indicate that the major bioactive constituents in PMTL are predominantly associated with beneficial health effects [23]. Pectin possesses a broader spectrum of health benefits relative to other prebiotics, including pronounced prebiotic properties. Notably, pectin constitutes the most abundant nutrient in PMTL, therefore we hypothesize that the dietary intake of PMTL may modulate the gut microbiota structure, thereby alleviating constipation [24,25,26,27]. However, studies on the application of PMTL for the management of functional constipation remain limited, to date [28].

In the present study, we investigated for the first time the preventive effects of PMTL ingestion on diphenoxylate-induced functional constipation in rats by analyzing constipation-related physiochemical parameters and gut microbiota composition. In addition, the morphology of the colon, the structure of colonic mucosa, and the status of colonic cell apoptosis were evaluated. Moreover, some neurotransmitters, inflammatory cytokines, aquaporins, and SCFAs were further measured to evaluate the potential mechanism of PMTL in relieving the experimental constipation. Collectively, these findings offer a theoretical foundation for the potential utilization of PMTL as a dietary supplement aimed at improving intestinal health.

## 2. Materials and Methods

### 2.1. Chemicals and Reagents

Dry *Premna microphylla* Turcz leaves (PMTL) were bought from Shaanxi Langao County Green Bamboo Food Co., Ltd. (Ankang, China). Carmine was purchased from Yuanye Biotechnology Co., Ltd. (Shanghai, China). Enzyme-linked immunosorbent assay (ELISA) kits of motilin (MTL), acetylcholine (ACH), somatostatin (SS), and substance P (SP) were purchased from Nanjing Jiancheng Bioengineering Institute (Nanjing, China). The primary antibodies Aquaporin 3 (AQP3) and Aquaporin 4 (AQP4) were purchased from Abcam (Cambridge, MA, USA). Reverse transcription kits, SYBR green-based quantitative PCR master mix, and primers for RT-qPCR assays were supplied by Sangon Biotech Co., Ltd. (Shanghai, China).

### 2.2. Chemical Composition Analysis of PMTL

The chemical composition of PMTL was determined according to the previous method [29,30]. Specifically, the total phenolic content was assayed according to the Folin–Ciocalteu method, with gallic acid as the standard. The results were expressed as milligrams of gallic acid equivalents per gram of dry weight (mg GAE/g). Similarly, the total flavonoid content was quantified using an aluminum chloride colorimetric assay, with rutin as the standard, and expressed as milligrams of rutin equivalents per gram of dry weight (mg RE/g). Total carbohydrates were measured by the phenol–sulfuric acid method using glucose as the standard, and the results are given as milligrams of glucose equivalents per gram (mg GE/g). Protein content was determined using the Kjeldahl method and converted to crude protein content using a factor of 6.25. Crude fat was extracted with petroleum ether (boiling point 60–90 °C) in a Soxhlet extractor for 6 h and reported as the percentage of fat relative to the dry weight of the sample.

### 2.3. Preparation of PMTL-Supplemented Feeds

PMTL powder was ground and sifted through 200 meshes and mixed with dietary feeds for experimental rats. The experimental diets were prepared by thoroughly mixing powdered PMTL with the standard rodent chow at concentrations of 5%, 10%, and 20% (*w*/*w*, g PMTL/100 g feed). The standard chow (provided by Xietong Feed Factory, Shanghai, China) contained a balanced mix of corn, wheat, fish meal, chicken meal, soybean meal, soybean oil, amino acids, vitamins, and minerals, with approximate nutritional values of 20% crude protein, 5% crude fat, and 55% carbohydrates. The mixed rat chow was first passed through a feed pellet machine (Qufu Huizhong Machinery Equipment Co., Ltd., Qufu, China) and then dried in a drying oven (Q/BKYY31-2000, Shanghai Yuejin Medical Instrument Factory, Shanghai, China) at 60 °C for 12 h. Finally, rat chow containing PMTL in different mass fractions (5%, 10%, and 20%) was obtained.

### 2.4. Animals and Experimental Design

**Figure 1:**
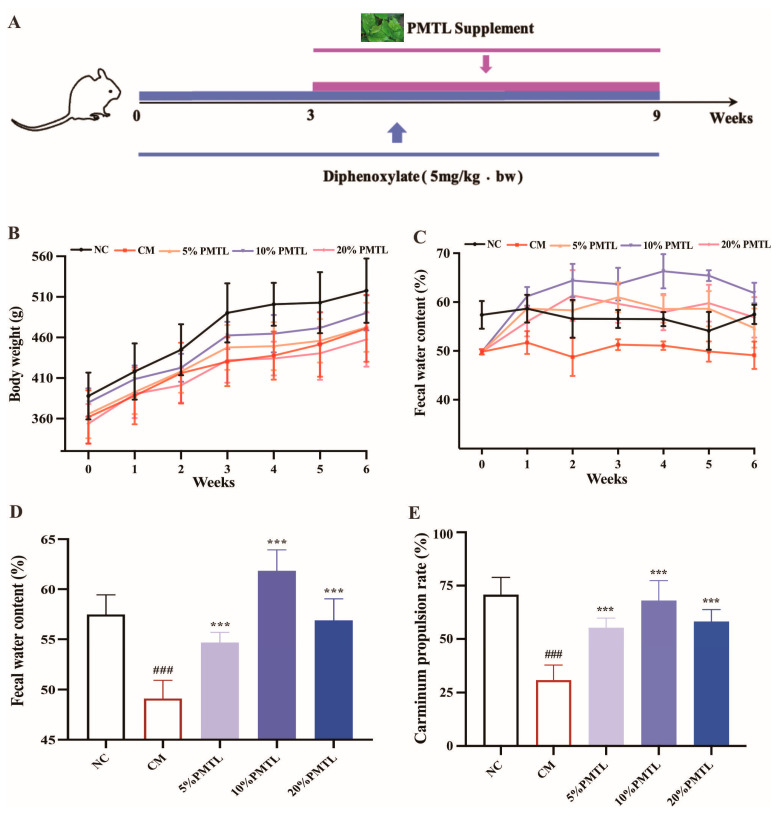
Body and fecal parameters of the rats treated with dietary *Premna microphylla* Turcz leaves (PMTL) for 6 weeks. (**A**) Experimental design of constipation induced by diphenoxylate in SD rats. (**B**) Body weight changes. (**C**) Changes in fecal water content. (**D**) Fecal water content of rats during the last week of the experiment. (**E**) Carmine propulsion rate in rat small intestine. (###) *p* < 0.001 different from the normal control (NC) rats. (***) *p* < 0.001, different from the constipation model (CM) rats.

Fifty male Sprague–Dawley (SD) rats, aged 4–6 weeks with an average body weight of 180 ± 10 g, were obtained from the Experimental Animal Center of the Fourth Military Medical University (Shaanxi, China). The animals were housed under standard laboratory conditions, maintained at 22 °C with 45–55% relative humidity and a 12 h light/dark cycle. Food and water were provided ad libitum throughout the acclimatization and experimental periods. After a week of adaptive feeding, all rats were randomly assigned to five groups (*n* = 10 per group): normal control (NC), constipation model (CM) induced by diphenoxylate, and three intervention groups receiving PMTL at doses of 5%, 10%, and 20%. The PMTL doses were determined based on cross-species conversion using body surface area normalization to ensure physiologically relevant exposure in the rat model [31]. The rats were induced with the classically functional constipation symptom according to the previous method with slight modification (Figure 1A) [32]. Briefly, rats in the NC group received 1 mL of normal saline, whereas those in the CM, 5% PMTL, 10% PMTL, and 20% PMTL groups were administered 1 mL of diphenoxylate solution (5 mg/kg body weight, bw). During weeks 1 to 3 of the experiments, all the rats were given standard rat chow. From weeks 4 to 9, the rats in 5% PMTL, 10% PMTL, and 20% PMTL groups were fed with chow supplemented by 5%, 10%, and 20% PMTL, respectively. Meanwhile, the rats in the NC and CM groups were fed with standard rat chow. After 9 weeks, all the rats were sacrificed, and their bloods were collected and centrifuged at 4 °C, 10,000× *g* for 15 min to obtain the serum samples and then placed in a −80 °C refrigerator. In addition, the intestines of the rats were also collected in a sterile centrifuge tube and stored at −80 °C for further analysis.

### 2.5. Defecation Test

At the end of each week from the third week, all the rats were placed in clean cages lined with absorbent paper, and all fresh feces excreted within 4 h were collected. The wet weight of feces was weighed and recorded. Then, the fresh feces were placed in a drying oven at 60 °C for 8 h, and the dry weight of feces was recorded after it was completely dried. The fecal water content was determined using the following equation: fecal water content (%) = [(wet weight − dry weight)/wet weight] × 100.

### 2.6. Test for Small Intestinal Propulsion Rate

The carmine propulsion rate of rats was determined on the last day of the animal experiments. After they were fasted for 12 h, all the rats were oral administrated with 1 mL carmine, and then 30 min later, the rats were sacrificed and dissected to obtain small intestines (from stomach to ileocecal junction). For each rat, the movement distance of carmine solution and the total length of the small intestine were measured on the background plate of photography. The small intestinal transit rate was calculated using the following formula: distance traveled by carmine/total length of small intestine) × 100 [33,34].

### 2.7. Histopathological Examination

For hematoxylin and eosin (H&E) staining, the colons of rats were fixed with 10% formalin solution and embedded in paraffin and cut into 5 μm thick slice. Then, all the sections were stained with H&E. For alcian blue staining, paraffin-embedded tissue sections were initially deparaffinized in xylene and subsequently rehydrated through a graded ethanol series. Tissue sections were rinsed with distilled water and finally stained using the alcian Blue Stain Kit (Genmed, Boston, CA, USA) according to the instructions. The apoptosis of colonic cells was detected by the Tunel Kit (MedChemExpress, Shanghai, China). Paraffin sections were dewaxed with xylene and then soaked in absolute ethanol, 90%, 80%, and 70% ethanol in turn, washed with PBS and incubated with proteinase K. After that, the samples were incubated with Tunel working solution in the dark, washed with PBS and finally added with fluorescence quenched sealing solution. The morphological characteristics of the colon sections were observed and photographed under an Axio Imager upright microscope (ZEISS, Axio Imager M2, Oberkochen, GER).

### 2.8. Measurement of Excitatory and Inhibitory Neurotransmitters

The serum neurotransmitters were determined with ELISA kits according to the kit instruction (Nanjing Jiancheng Bioengineering Institute, Nanjing, China). In addition, the colons of rats were thawed at room temperature, and then 100 mg of colons were weighed and placed in a centrifuge tube filled with 0.9 mL PBS. The colons were homogenized at −10 °C and then centrifuged at 4 °C and 10,000× *g* for 15 min to obtain the supernatant. Neurotransmitter levels in the colon homogenates were measured following the instructions provided with the commercial ELISA kits.

### 2.9. Western Blot Determination

Western blot analysis was conducted following a previously established protocol [35]. Total proteins from the colon tissue were extracted with RIPA lysis buffer containing PMSF (1:100) and phosphatase inhibitors (1:50), and the protein concentration was determined by BCA assay. After that, the loading buffer was added into each sample and boiled at 100 °C for 10 min to denature the proteins, and then all the denatured samples were frozen separately and stored in ultra-low temperature refrigerator at −80 °C. Sample proteins from each group were separated using prefabricated 10% stain-free gels, transferred to a PVDF membranes, and sealed in 5% bovine serum albumin. Subsequently, the PVDF membranes were incubated with primary antibodies overnight at 4 °C. The following day, after washing three times, the membranes were incubated with corresponding secondary antibodies for one hour at room temperature. Western blot images were acquired using a Bio-Rad ChemiDoc imaging system (Bio-Rad Laboratories, Hercules, CA, USA), and the gray values of each protein band were quantified with ImageJ software (2.0.0).

### 2.10. RT-qPCR Analysis

All of the RNA of the colon was extracted by reagent Trizol, and then reversely transcribed into double-stranded cDNA according to the instructions of the high-capacity cDNA reverse transcription kit. The target genes were amplified with SYBR green mix, and its fluorescence signal was detected by PCR instruments (Bio-Rad Laboratories, Hercules, CA, USA) *Gapdh* was used as an internal reference gene in this experiment. The primer sequences of all genes can be found in Table 1.

### 2.11. Gut Microbiota Analysis

The DNA from fecal samples of the rat colon was extracted using the cetyl trimethyl ammonium bromide (CTAB) method. The relative purity of extracted DNA was determined by 1% agarose gel electrophoresis, and the concentration was determined by a NanoDrop 2000 UV-vis spectrophotometer (ThermoFisher Scientific, Waltham, MA, USA). After that, the V3–V4 region of bacterial 16S rRNA genes was amplified by primers 338F (ACTCCTACGGGAGGCAGCAG) and 806R (GGACTACHVGGGTWTCTAAT) for 30 cycles with the conditions described previously [36]. High-throughput sequencing of the bacterial 16S rRNA gene was performed on Illumina MiSeq PE250 platform (Illumina, San Diego, CA, USA). Raw paired-end reads were demultiplexed, subjected to quality filtering, and merged based on overlapping regions to generate high-quality sequences [37]. Denoising of these sequences was carried out to derive amplicon sequence variants (ASVs) along with their abundance profiles. Subsequent analyses, including taxonomic classification, community diversity assessment, and differential abundance testing, were conducted using these ASV data. For taxonomic assignment, sequences were analyzed with the RDP Classifier algorithm against the SILVA SSU123 16S rRNA database, applying a confidence threshold of 70% to ensure classification accuracy.

### 2.12. GC-MS Analysis for SCFAs

The analysis of short-chain fatty acids (SCFAs) was based on the previous method [38]. An amount of 100 mg of colon contents from rats were extracted with 1 mL of 5% phosphate and centrifuged at 4 °C, 10,000× *g* for 10 min. The supernatant was collected and subjected to extraction using 500 μL of ethyl acetate. Following this, the organic phase, which contained the SCFAs, was isolated and passed through a 0.22 μm PVDF organic filter membrane prior to analysis. Gas chromatography–mass spectrometer (GC-MS) with an Agilent DB-WAX capillary column (30 m × 0.25 mm ID × 0.25 μm, Agilent Technologies, Santa Clara, CA, USA) was used to separate the SCFAs extractives. Quantification of SCFAs in rat colonic contents was performed by comparing their levels to those of authentic chemical standards.

### 2.13. Data Analysis

Total data are presented as mean ± standard deviation (SD). GraphPad Prism (Version 9.0) was used for Student’s *t*-test analysis of the two groups, and one-way ANOVA was performed for multiple groups to analyze the statistical differences between the groups. *p* < 0.05 was considered statistically significant. All graphical representations were generated using GraphPad Prism software.

## 3. Results

### 3.1. Chemical Composition of PMTL and Its Effects on Body Weight and Fecal Water Content

By measuring the chemical composition of *Premna microphylla Turcz* leaves (PMTL) in dried powder form, it was determined that the contents of total polyphenols, total flavonoids, total carbohydrates, total soluble proteins, and total fats in PMTL were 116.8 ± 1.3 mg GAE/g dw, 150.6 ± 4.1 mg RE/g dw, 459.9 ± 3.2 mg GE/g dw, 41.3 ± 0.37% (*w*/*w*), and 41.6 ± 0.19% (*w*/*w*), respectively (Table 2), indicating that PMTL is particularly rich in total carbohydrates, which are a major component of soluble dietary fiber and may include compounds such as pectin. As can be seen from Figure 1B, the weight of all the rats kept rising during the experimental period, and the increase in the weight of the NC rats was the most significant, followed by the 10% PMTL group rats. Figure 1C showed that the fecal water content of rats in the NC group was maintained at about 58.0% during the whole experimental cycle, while the fecal water content of rats in diphenoxylate-induced constipation model (CM) group was maintained at about 50%, which was lower than that in the NC group. However, the intake of 10% PMTL significantly increased the fecal water content of diphenoxylate-induced CM rats. As can be seen from Figure 1D, the final fecal water content of the rats in CM group was significantly lower than that in the NC group (*p* < 0.01). Interestingly, compared with the CM group, the final fecal water contents of the CM rats ingesting 5%, 10%, and 20% PMTL were all significantly increased (*p* < 0.01), and 10% PMTL supplementation had the most obvious effect on improving final fecal water content of the CM rats. These results proved that PMTL could improve fecal water content in the tested constipated rats by improving intestinal water secretion and reabsorption.

### 3.2. PMTL Promoted Carmine Propulsion Rate in Constipated Rats

As shown in Figure 1E, diphenoxylate treatment led to a significant decrease in the carmine propulsion rate of rats in the CM group when compared to the NC group (Figure 1E, *p* < 0.01), suggesting that the diphenoxylate-induced constipation model was successfully established. Interestingly, the carmine propulsion rate of the rats in 5%, 10%, and 20% PMTL groups was significantly higher than that in the CM group rats (*p* < 0.01), especially the rats in the 10% PMTL group, showing the highest carmine propulsion rate among all the PMTL treatment groups. The results indicated that PMTL markedly enhanced the intestinal propulsion rate in constipated model rats, suggesting its potential to stimulate gastrointestinal motility.

### 3.3. PMTL Altered Colonic Histological Structure of Constipated Rats

**Figure 2:**
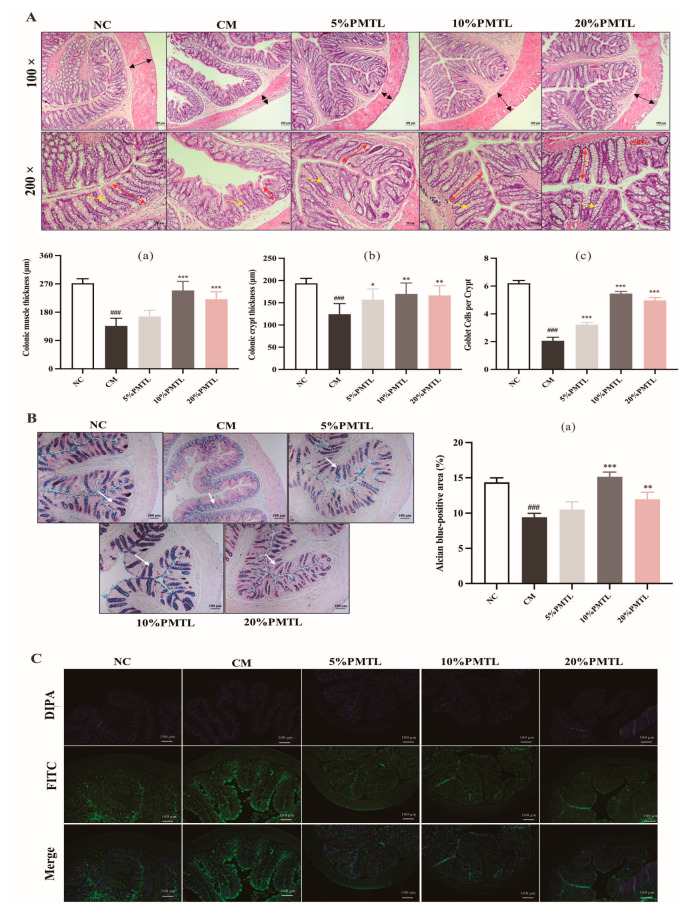
Colonic morphology and colonic cell apoptosis of the diphenoxylate-induced CM rats treated with dietary PMTL for 6 weeks. (**A**) H&E section staining of the colon: (a) colonic muscle thickness; (b) colonic crypt thickness; (c) goblet cell per crypt; (**B**) alcian blue-positive staining of the colon: (a) alcian blue-positive area; (**C**) image of colonic cell apoptosis in rats determined by the TUNEL kit (MedChemExpress, Shanghai, China). (###) *p* < 0.001 different from the NC rats. (*) *p* < 0.05, (**) *p* < 0.01, and (***) *p* < 0.001, different from the CM rats.

The H&E staining images of the colon of rats are shown in Figure 2A. In comparison with the NC group, the structure of goblet cells of rats in CM group was not clear, and the number of goblet cells per crypt was significantly decreased (*p* < 0.01). In addition, the colonic muscle thickness and crypt thickness of rats in the CM group were significantly reduced as compared with the rats in the NC group. However, compared with the CM group, the colonic muscle thickness of the rats in the 10% and 20% PMTL groups was significantly increased (*p* < 0.01), while the colonic crypt length of the rats in all PMTL consumption groups were dramatically elevated (*p* < 0.05). Furthermore, quantitative analysis revealed that PMTL administration, particularly at the 10% dose, markedly increased the number of goblet cells per crypt compared to the CM group (*p* < 0.01), demonstrating a dose-dependent restorative effect. In addition, the colonic epithelium and smooth muscle layer structure of PMTL-treated rats were intact, and the boundary between crypt structure and goblet cells was clear in contrast to the CM group. The results of H&E staining images showed that PMTL could not only effectively increase colonic muscle and crypt thickness and restore goblet cell numbers but also improve goblet cell morphology and volume in colons of constipated rats.

As can be seen from alcian blue staining images, the acid mucin area of crypt cells in the distal colon tissue of rats in the CM group was significantly decreased compared to the NC group (Figure 2B, *p* < 0.01). However, after supplementation of PMTL, the area of acid mucin of rats in all the PMTL groups had a significant increase, and the structure and alcian blue-positive area of the rats in the 10% PMTL group were even similar to that in the NC group. The alcian blue staining results showed that PMTL could stimulate the secretion of acidic mucus in the colon and protect colon tissues of rats against constipation. Figure 2C illustrates apoptosis in colonic cells, where stronger fluorescence intensity corresponds to more severe apoptosis. Compared to the NC rats, the CM rats exhibited a significant increase in apoptotic cell positivity. However, the administration of PMTL markedly reduced the fluorescence intensity in the colon cells of the CM rats, with the 10% PMTL group showing the most pronounced effect. These results indicate that PMTL can suppress colonic cell apoptosis and help maintain normal colonic morphology in constipated rats.

### 3.4. PMTL Regulated Excitatory and Inhibitory Neurotransmitters in Rats

**Figure 3:**
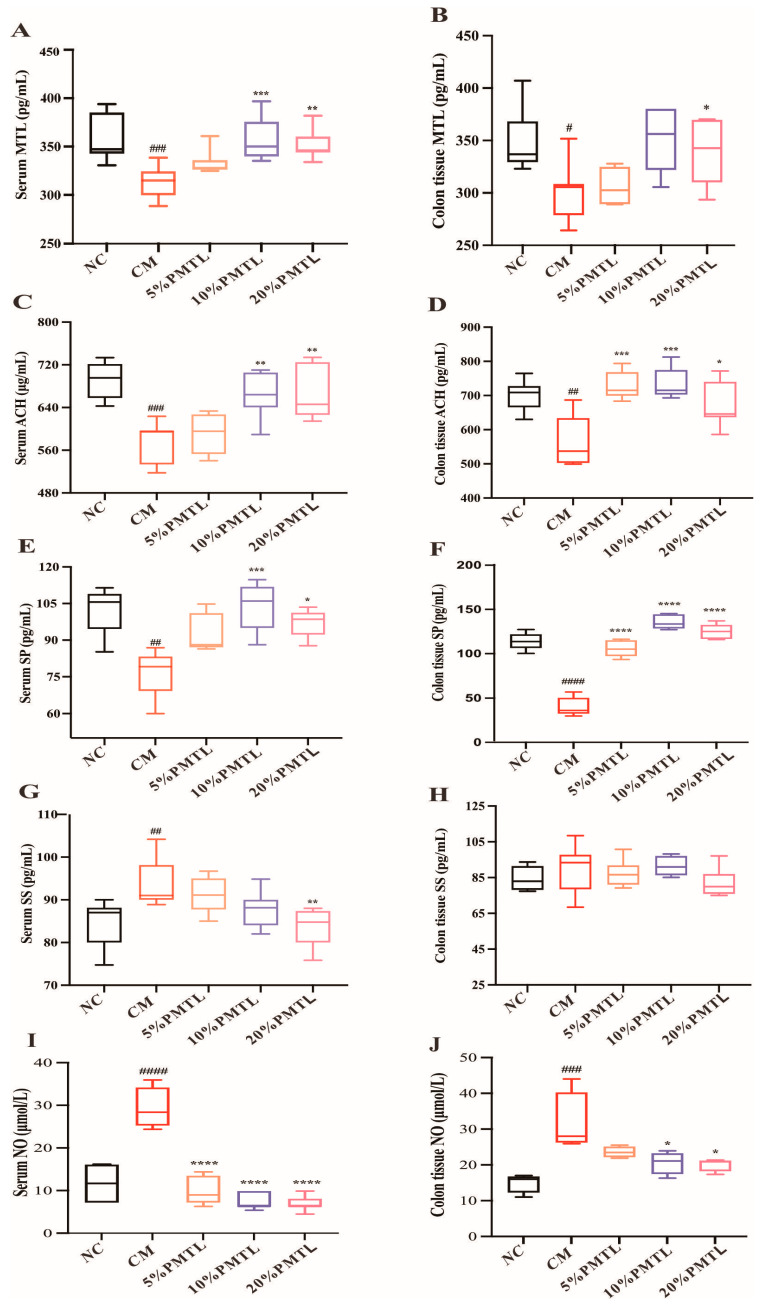
Effects of PMTL supplementation on excitatory and inhibitory neurotransmitters in the serum and the colon of the CM rats. Serum MTL (**A**), ACH (**C**), SP (**E**), SS (**G**), NO (**I**); colon tissue MTL (**B**), ACH (**D**), SP (**F**), SS (**H**), NO (**J**). (#) *p* < 0.05, (##) *p* < 0.01, (###)and (####) *p* < 0.001 different from the NC rats. (*) *p* < 0.05, (**) *p*< 0.01, (***) and (****) *p* < 0.001, different from the CM rats.

As excitatory neurotransmitters, serum and colonic tissue levels of motilin (MTL), acetylcholine (ACH), and substance P (SP) were significantly lower in the CM group compared to the NC group (Figure 3A–F, *p* < 0.05). However, compared with the CM group, the 10% PMTL supplement showed the most obvious effect on the elevation in the serum MTL, ACH, and SP levels with an increase by 12.2%, 16.2%, and 35.3% (Figure 3A,C,E, *p* < 0.05), respectively, followed by the 20% PMTL consumption in rats. Similarly, the tested excitatory neurotransmitters including MTL, ACH, and SP were also improved in the colon tissue of PMTL-treated rats in comparison with the CM rats (Figure 3B,D,F, *p* < 0.05), where MTL was increased by 11.6% in the 20% PMTL group, ACH was increased by 30.6% in the 10% PMTL group, and SP was increased by 236.4% in the 10% PMTL group. The findings indicate that the administration of PMTL led to an elevation in the concentrations of excitatory neurotransmitters, including MTL, ACH, and SP, in rats with constipation.

Furthermore, the serum inhibitory neurotransmitter somatostatin (SS) of rats in the CM group were effectively increased, relative to the NC group (Figure 3G, *p* < 0.01), while such an effect was not observed in the colon tissue of rats (Figure 3H, *p* > 0.05). However, the 20% PMTL treatment resulted in a 11.2% reduction in serum SS concentrations (*p* < 0.01). The levels of nitric oxide (NO), another inhibitory neurotransmitter, of the colon and the serum in the CM rats were greatly elevated by 109.7% and 150.9%, respectively, as compared with the NC group. Interestingly, the elevated serum and colonic NO levels were effectively lowered following treatment of the CM rats with 10% or 20% PMTL (Figure 3I,J, *p* < 0.01). The present results suggested that PMTL could inhibit the secretion of inhibitory neurotransmitters (SS and NO) in diphenoxylate-induced constipation rats.

### 3.5. Effects of PMTL on Expressions of Aquaporins and Inflammatory Factors in Rats

**Figure 4:**
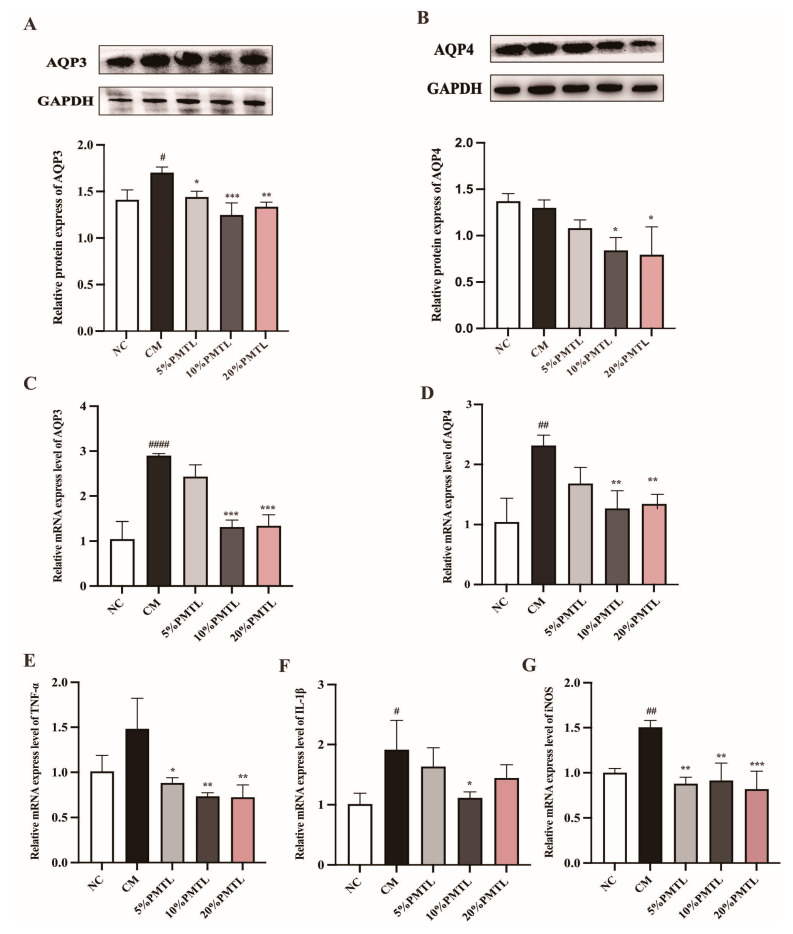
Effects of PMTL ingestion on protein and mRNA expression in the colon of the CM rats. Levels of the protein expression of (**A**) AQP3 and (**B**) AQP4; levels of the mRNA expression of (**C**) AQP3, (**D**) AQP4, (**E**) THF-α, (**F**) IL-1β, and (**G**) iNOS. (#) *p* < 0.05, (##) *p* < 0.01, and (####) *p* < 0.001 different from the NC rats. (*) *p* < 0.05, (**) *p* < 0.01, and (***) *p* < 0.001, different from the CM rats.

To elucidate the mechanism by which PMTL regulates the water-holding capacity of the colon, the expression levels of aquaporins AQP3 and AQP4 were assessed using Western blot and RT-qPCR, respectively. As described in Figure 4A–D, the colonic AQP3 and AQP4 proteins and gene expressions of the CM rats were obviously greater than those of the NC rats. However, supplementation of 10% and 20% PMTL in the constipated rats significantly reduced the colonic AQP3 and AQP4 protein and their genes expressions (*p* < 0.05). In addition, the expressions of inflammatory genes of *Tnfα*, *IL1b*, and *Nos2* are shown in Figure 4E–G. Except for *Tnfα*, the levels of *IL1b* and *Nos2* genes in the colon tissue of the CM rats were markedly increased as compared with the NC rats (*p* < 0.05). As expected, PMTL intake significantly reduced the expressions of inflammatory factor genes in the colon of the constipated rat at all the tested dosages. These results suggest that PMTL may alleviate constipation symptoms in rats to some extent by reducing the colonic expressions of aquaporins-3 and aquaporins-4, as well as inflammatory factors in rats.

### 3.6. PMTL Alleviated Gut Microbiota Disturbance in Constipated Rats

**Figure 5:**
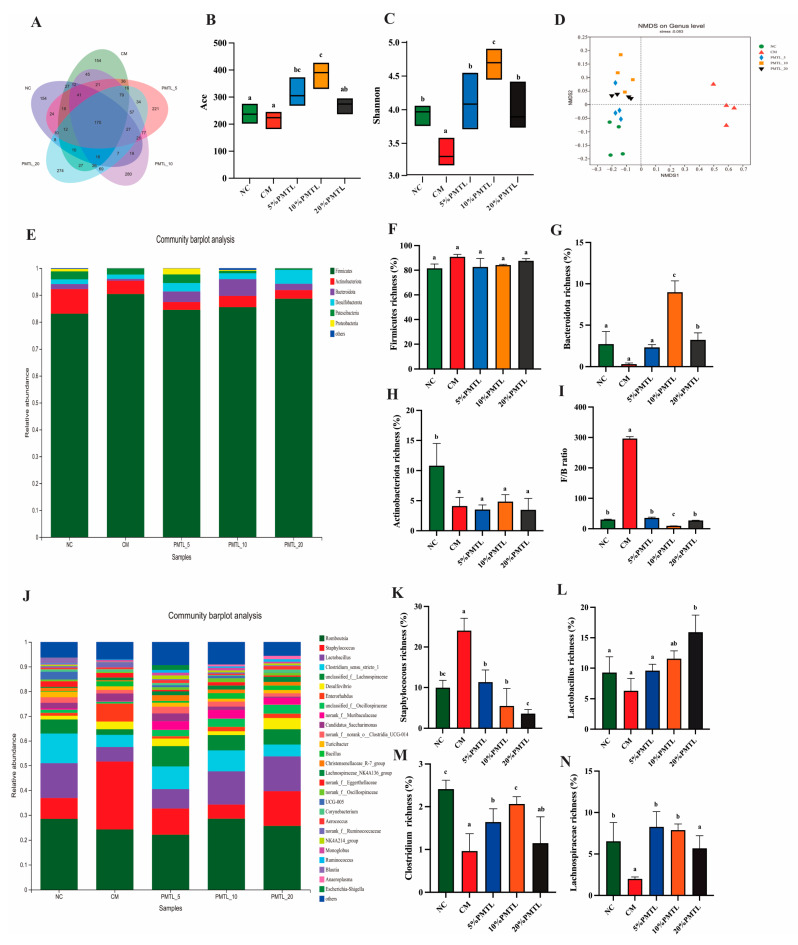
Effects of PMTL on intestinal microbiota in the colon of the CM rats. (**A**) Diversity of intestinal microbiota at the operational taxonomic unit (OTU) level. (**B**) Ace index analysis of the richness and (**C**) Shannon index analysis of the richness. (**D**) Non-metric multidimensional scaling (NMDS). (**E**) Distribution of gut microbiota at phylum level. (**F**) *Firmicutes* richness. (**G**) *Bacteroidota* richness. (**H**) *F*/*B* ratio. (**I**) *Actinobacteriota* richness. (**J**) Distribution of gut microbiota at genus level. (**K**) *Staphylocoocus* richness. (**L**) *Lactobacillus* richness. (**M**) *Lachnospiraceae* richness. (**N**) *Clostridium* richness. (*p* < 0.05) significant differences are indicated by different letters.

As can be seen from Figure 5A, PMTL supplementation resulted in an increase in the diversity of gut microbiota in the CM rats. As shown in Figure 5B,C, compared with the NC group, diphenoxylate caused significant reduction in the ACE and Shannon indices of gut microbiota, whereas PMTL treatment tended to increase the richness and diversity of gut microbiota in constipated rat to a certain extent (*p* < 0.05). Nursing Minimum Dataset (NMDS) results suggested that diphenoxylate-caused constipation could damage the intestinal microbiota structure of rats, while the gut microbiota of rats in PMTL groups was closer to that of rats in the NC group (Figure 5D).

Phylum level analysis showed that the intestinal bacteria of rats mainly included *Firmicutes, Actinobacteriota*, *Bacteroidota*, *Desulfobacterota,* and *Patescibacteria* (Figure 5E). As shown in Figure 5F, no statistically significant differences were observed in the relative abundance of Firmicutes across the experimental groups (*p* > 0.05). In contrast, rats treated with 10% PMTL exhibited a marked increase in the abundance of *Bacteroidota* compared to the CM group (Figure 5G, 8.78 ± 0.8, *p* < 0.01). As shown in Figure 5H, the *Firmicutes*/*Bacteroidota* (*F*/*B*) ratio of the CM rats was significantly higher than that of the NC rats (266.2 ± 3.8, *p* < 0.05). In comparison with the CM group, dietary consumption of PMTL at all the tested doses dramatically decreased the *F*/*B* ratio of the constipated rats (*p* < 0.05), in which ingestion of 10% PMTL exerted the most obvious effect (*p* < 0.01). Similarly, the abundance of *Actinobacteriota* was significantly decreased in the CM rats (*p* < 0.05 vs. the NC rats), while PMTL intake was not greatly altered in its abundance relative to the CM rats (Figure 5I, *p* > 0.05). At the genus level, compared with the NC group, *Staphylococcus* richness was increased, while the richness of *Lactbacillus*, *Lachnospiraceae*, and *Clostridium* were decreased in the CM group (Figure 5K–N). In contrast to the CM group, the abundances of *Staphylococcus*, *Lactbacillus*, *Lachnospiraceae*, and *Clostridium* in all the PMTL-treated groups were remarkably elevated (*p* > 0.05), similar to the NC group. Overall, the relative abundance of microbial composition at phylum level and genus level indicated that the microbial composition of diphenoxylate-induced constipated rats was significantly changed, and the ingestion of PMTL could regulate the microbial composition of constipated rats to move closer to the NC rats.

### 3.7. Effects of PMTL Supplementation on Colonic SCFAs in Rats

**Figure 6:**
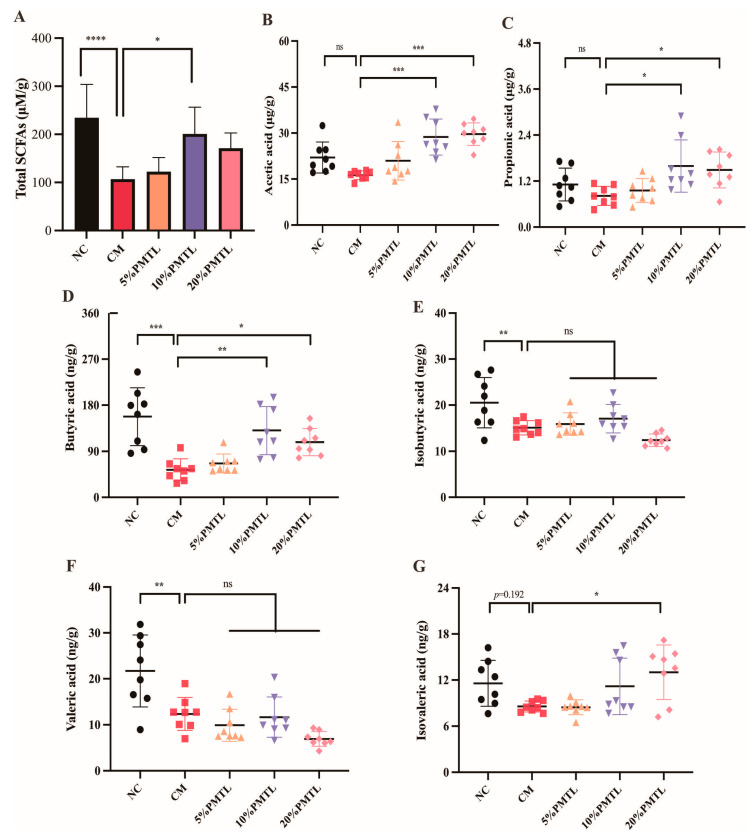
Effects of PMTL intake on SCFAs in the colon of the CM rats. Levels of the (**A**) total SCFAs, (**B**) acetic acid, (**C**) propionic acid, (**D**) butyric acid, (**E**) isobutyric acid, (**F**) valeric acid, and (**G**) isovaleric acid. ns indicates no significant difference, (*) *p* < 0.05, (**) *p* < 0.01, (***) and (****) *p* < 0.001, different from the CM rats.

**Figure 7:**
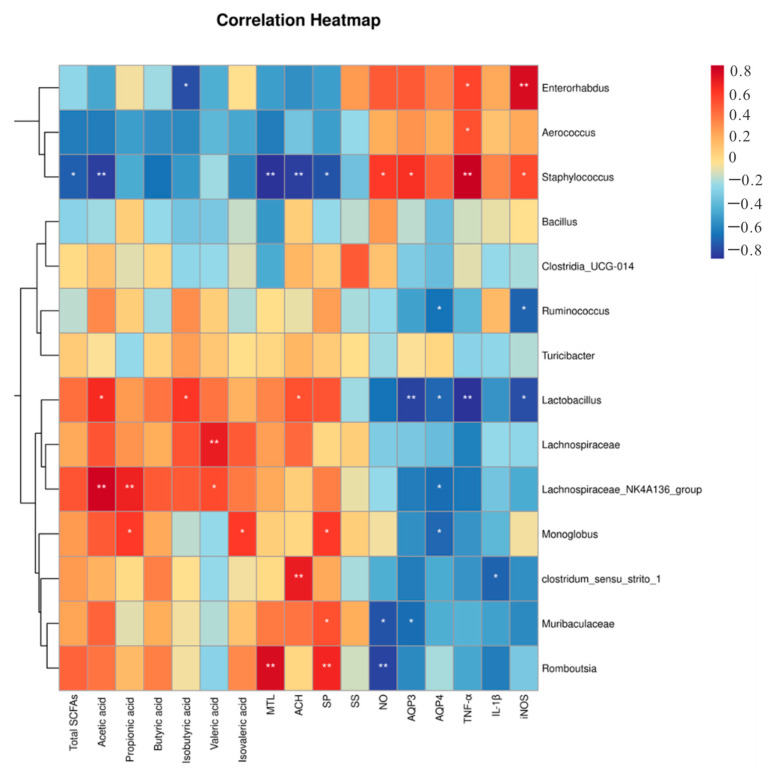
Spearman’s correlation was used to analyze the correlation between intestinal microbiota and short-chain fatty acids (SCFAs), neurotransmitters, AQPs, and inflammatory cytokines. (*) *p* < 0.05, (**) *p* < 0.01 indicate the association significant, respectively.

Short-chain fatty acids (SCFAs) are important metabolites of dietary fiber fermented by gut microorganisms, and considered to be important metabolites [39,40]. As shown in Figure 6A, the total SCFA levels in the colon of rats in the NC group and the CM group were 234.6 ± 60.9 μM/g and 106.9 ± 26.3 μM/g, respectively. It can be found that the total SCFA level in the colon of rats in the CM group decreased by 54.4% compared with the NC group (*p* < 0.01). Interestingly, 10% PMTL supplementation in the CM rats greatly elevated the colonic total SCFA concentrations (*p* < 0.05). As shown in Figure 6B–F, the levels of butyric acid, isobutyric acid, and valeric acid in constipated rats were 65.9%, 26.6%, and 43.1% lower than those in the NC rats (*p* < 0.05). The levels of acetic acid and butyric acid in the CM rats given 10% PMTL were 28.7 ± 1.9 μg/g and 130.5 ± 15.3 ng/g, respectively, which increased by 142.7% and 100.4% compared with the model group (*p* < 0.05). Additionally, Spearman’s correlation analysis was employed to assess the relationships among gut microbiota composition and levels of SCFAs, neurotransmitters, aquaporins, and inflammatory cytokines. Figure 7 showed that the acetic acid had a positive correlation with *Lactobacillus*, *Lachnospiraceae_NK4A136_group* (r = 0.623, *p* < 0.05; r = 0.767, *p* < 0.05). Moreover, the levels of NO, AQP3, THF-α, and iNOS exhibited a positive correlation with *Staphylococcus* (r = 0.579, *p* < 0.05; r = 0.609, *p* < 0.05; r = 0.778, *p* < 0.01; r = 0.541, *p* < 0.05).

## 4. Discussion

The classic drug diphenoxylate exerts a direct inhibitory effect on intestinal smooth muscle contraction, thereby slowing peristalsis and resulting in constipation [34,41]. Herein, fecal water content and carmine propulsion rate were, therefore, used to evaluate the mitigatory effects of PMTL on diphenoxylate-induced constipation. Stool and defecation characteristics are thought to be a direct reflection of constipation [42]. In the present study, constipation induced by diphenoxylate resulted in the decrease in fecal water content, carmine propulsion rate, and body weight of the tested CM rats; these results confirm that the rat constipation model was successfully established through diphenoxylate administration. Supplementation of PMTL merged into the animal chow increased the body weight, fecal water content, and carmine propulsion rate of the constipated rats to different degrees, indicating that PMTL exerted an excellent effect in relieving the diphenoxylate-caused constipation (Figure 1B–E). This observed effect may be associated with the presence of prebiotics such as pectin or dietary fiber in PMTL.

It is well known that the colon plays a significant physiological role in the excretion of feces, and its physiological structure is considered to be an important index for evaluating constipation system [43]. Previous studies have confirmed that constipation can cause pathological changes in the physiological microbiotic structure of the colon, such as abnormal secretion of colonic mucus cells and thin colonic muscles. Consistent with previous studies, the results of this study demonstrated that the colonic muscle thickness and crypt thickness of constipated rats were significantly thinner than the normal rats [44]. However, consumption of PMTL in rats was beneficial to increase the muscle and crypt thickness of the colon and improve the secretion of mucus cells, and the colonic morphology of PMTL-treated rats was close to that of normal rats (Figure 2A–C). The anti-constipate effect of PMTL appears to be mediated through the modulation of inflammatory pathways. Treatment with PMTL significantly attenuated the constipation-associated upregulation of key pro-inflammatory mediators, including TNF-α, IL-1β, and inducible nitric oxide synthase (iNOS) (*p* < 0.05, Figure 4E–G). The suppression of iNOS is of particular mechanistic importance, as it likely reduces the excessive production of nitric oxide-derived reactive nitrogen species, thereby alleviating nitrosative stress and its detrimental effects on colonic smooth muscle motility [45]. Concurrently, the reduction in TNF-α and IL-1β may contribute to the restoration of epithelial barrier integrity and normalization of mucosal water transport [46]. These findings suggest that the therapeutic efficacy of PMTL extends beyond mere laxation and includes the resolution of underlying mucosal inflammation, which represents a critical pathophysiological component of chronic constipation. Based on these results, we speculate that PMTL may improve colonic morphology and physiological function in constipated rats by reducing the expression of inflammatory cytokines. It should also be noted that polyphenols present in PMTL could contribute to these anti-inflammatory effects, suggesting a potential multi-component mechanism behind the observed therapeutic outcomes.

Dysregulated neurotransmitter release may contribute to impaired intestinal smooth muscle contraction, representing a key etiological factor in the development of constipation [47]. Neurotransmitters including MTL, ACH, SP, SS, and NO play a crucial role in the regulation of gastrointestinal motility [48]. MTL not only affects the transport of water and electrolytes but also promotes gastrointestinal peristalsis to stimulate the secretion of hydrochloric acid, pancreatic juices, and biles by gastrointestinal cells [15]. ACH is the main transmitter in the enteric nervous system, which can regulate bowel function by combining with its receptors [15]. SP is a neuropeptide widely distributed in fine nerve fibers, which can prick Cajal intestinal cells and act as pacemakers for intestinal smooth muscle contraction and gastrointestinal peristalsis [49,50]. SS and NO are widely recognized as inhibitory neurotransmitters that play a regulatory role in gut function, including the suppression of digestive enzyme secretion and the inhibition of intestinal motility [51]. In general, under the induction of diphenoxylate, the body may produce more inhibitory neurotransmitters (SS and NO) and few excitatory neurotransmitters (MTL, ACH, and SP) to cause constipation. In this study, it was shown that the levels of SS and NO were increased and the levels of MTL, ACH, and SP were decreased in the serum and the colon of CM rats caused by diphenoxylate (Figure 3). However, these alterations in inhibitory and excitatory neurotransmitters were dramatically reversed by PMTL ingestion in the CM rats. Concomitantly, SP levels were increased markedly (>200%) following PMTL intervention, indicating potent neuroexcitatory effects. Although the magnitude of this change may be influenced by pre-treatment depletion in the constipation model or potential nonlinearity in quantification at high concentrations, the upward trend remains robust and biologically indicative. These findings indicate that PMTL supplementation could enhance intestinal motility in rats by modulating the equilibrium between inhibitory and excitatory neurotransmitters. Consistent with previous reports, our findings further support the crucial role of the equilibrium between excitatory and inhibitory neurotransmitters in regulating intestinal motility [47].

It is noteworthy that PMTL rich in soluble pectin, significantly elevated fecal water content in constipated CM rats when supplemented at 5–20% in chow. Notably, its dose–response was non-linear, with 10% PMTL being more effective than 20% (Figure 1D). We speculate that excessive dietary fiber may slow gastrointestinal fermentation, thereby impairing fecal water content regulation in the CM rats and weakening the efficacy of the 20% dose [52]. In addition, AQPs play an important role in the intestinal water transport system, which can increase or decrease intestinal water absorption or secretion, thereby regulating intestinal fecal parameters [53]. Emerging evidence indicates that dysregulated expression of colonic aquaporins (e.g., AQP3 and AQP4) may lead to excessive colonic water absorption and/or reduced secretion of intestinal fluid, thereby contributing to the pathogenesis of constipation [54,55,56]. AQP3 is predominantly localized to the mucosal epithelial cells of the colon, where it is believed to critically regulate water reabsorption processes [56]. AQP4 has been demonstrated to play a critical role in mediating transepithelial water transport in response to osmotic gradients [57]. In our study, the CM rats expressed colonic AQP3 and AQP4 at higher levels than the untreated NC rats, while dietary PMTL intake of the CM rats inhibited this change caused by constipation, suggesting that PMTL might restrict the absorption of colon water to alleviate constipation symptoms by reducing the expressions of AQP4 and AQP3 in rats (Figure 4A–D).

It is widely recognized that the imbalance of gut microbiota is one of the main factors leading to constipation. Previous studies have reported a strong link of microecological flora with gastrointestinal diseases which can be improved by regulating intestinal flora [44,58]. Consistent with the established mechanisms of drug-induced intestinal ecological dysregulation, our research results indicate that diphenoxate disrupts the homeostasis of colonic microbiota, and this interference is significantly reversed by PMTL intervention. This recovery may selectively promote the recovery of symbiotic or beneficial taxa by providing fermentable substrates [59]. Taxonomic analysis indicated that the administration of PMTL restored the abundance of several bacterial genera—including *Staphylococcus*, *Lactobacillus*, and *Lachnospiraceae*—that were altered in constipated rats. Notably, the abundance of several potential probiotic bacteria, including *Lactobacillus*, was markedly increased following PMTL treatment in constipated rats (Figure 5L) [60,61]. *Lactobacillus* as a common probiotic can promote the relief of constipation symptoms by regulating intestinal microbiota, promoting the secretion of anti-inflammatory factors, and improving the metabolism of bile acids, vitamins, and ascorbic acid [62]. *Lachnospiraceae NK4A136Group* are important producers of SCFAs for maintaining intestinal health [63]. *Aerococcus* is prone to cause inflammation in the intestine and *Staphylococcus* is a Gram-positive anaerobe that can cause intestinal infections [64,65]. On the contrary, other harmful bacteria such as *Staphylococcus* and *Aerococcus* are reduced in the constipated rat by PMTL treatment. Figure 7 shows a positive correlation between *Aerococcus* and the gene levels of inflammatory factor TNF-α, and the abundance of *Staphylococcus* is negatively correlated with the content of excitatory neurotransmitters (MTL, ACH, and SP), and positively correlated with inhibitory neurotransmitter (NO), AQP3, and inflammatory factors (THF-α, iNOS). The obtained results indicate that PMTL alleviates constipation symptoms, potentially through enhancing the proliferation of beneficial intestinal bacteria while suppressing pathogenic bacterial populations. Additionally, the polyphenolic compounds in PMTL are also likely to participate in the regulation of gut microbiota, further supporting its prebiotic function. SCFAs as key metabolites derived from gut microbiota play a crucial role in alleviating constipation by stimulating intestinal peristalsis, increasing luminal osmotic pressure and enhancing water absorption [66]. In constipated rats, PMTL treatment significantly elevated fecal SCFA levels—particularly acetic acid and butyrate—restoring them to a physiologically beneficial range. This restoration not only counteracted constipation-associated deficits but also reached concentrations considered optimal for colonic health, including anti-inflammatory effects and the promotion of normal intestinal function. The non-linear dose–response relationship was observed, in which the 10% PMTL dose showed better efficacy than the 20% dose. This may result from excessive fermentable fiber intake, which can lead to adverse effects—including gas production, bloating, and microbial changes—that counteract the benefits of SCFAs, highlighting the importance of optimal rather than maximal dosing [67]. Correlation analysis showed that *Lachnospiraceae NK4A136Group* was significantly and positively correlated with acetic acid, propionic acid, and valeric acid. *Lactobacillus* was markedly and positively associated with acetic acid and isobutyric acid. Mechanistically, the downregulation of pro-inflammatory factors (IL-1β, iNOS) and aquaporins (AQP3, AQP4) was likely mediated by SCFAs derived from PMTL pectin fermentation. Butyrate, in particular, activates GPCRs (e.g., GPR41/43/109a), suppressing NF-κB signaling and subsequent inflammation. The attenuated inflammatory milieu further normalizes water transport, leading to reduced AQP3/AQP4 expression. These findings suggested that PMTL might alleviate constipation via the microbiota–SCFAs–inflammation axis, restoring intestinal homeostasis.

## 5. Conclusions

In conclusion, this study demonstrates that PMTL effectively alleviates diphenoxylate-induced constipation in rats through multi-faceted mechanisms. The most critical findings include the significant enhancement of intestinal motility, restoration of gut barrier integrity, and a notable reshaping of the gut microbiota composition, which collectively led to favorable changes in associated metabolites and neurotransmitters.

To enhance the translational value of this study, future work should (1) investigate synergistic effects between PMTL and probiotics/prebiotics; (2) optimize the structure of PMTL-derived polysaccharides to improve their bioavailability; and (3) apply metagenomic sequencing with PICRUSt2/KEGG analysis to functionally link gut microbiota remodeling to PMTL’s physiological effects.

## Figures and Tables

**Table 1:** Primers used for real-time quantitative PCR.

Gene	Forward Primer	Reverse Primer
*Aqp3*	TGGACCTCGCCTTTTCACT	GACACCACCAATGGAACCCA
*Aqp4*	ACCTGTGATAGCACCTTGCCC	CAGACGCCTTTGAAAGCCAC
*Gapdh*	GCATCTTCTTGTGCAGTGCC	GGTAACCAGGCGCCGATAC
*Tnfα*	GACCCTCAGACTCAGATCATCCTTCT	ACGCTGGCTCAGCCACTC
*Il1b*	CTCCATGAGCTTTGTACAAGG	TGCTGATGTACCAGTTGGGG
*Nos2*	AGAGAGATCGGGTTCACA	CACAGAACTGAGGGTACA

**Table 2:** Composition analysis of polyphenols, flavonoids, carbohydrates, soluble proteins, and fats in *Premna microphylla Turcz* leaves (PMTL).

Total Component	Regression Curve	R^2^	Content (Mean ± SD)
polyphenols	y = 38.1x + 0.0502	0.9992	116.8 ± 1.3 mg GAE/g dw
flavonoids	y = 6.718x − 0.2861	0.9985	150.6 ± 4.1 mg RE/g dw
carbohydrates	y = 2.3629x + 0.0405	0.9996	459.9 ± 3.2 mg GE/g dw
soluble proteins			41.3 ± 0.37% (*w*/*w*)
fats			41.6 ± 0.19% (*w*/*w*)

Note: The components listed are not mutually exclusive mass fractions. Bioactive compounds (polyphenols and flavonoids) are secondary metabolites biosynthesized within the plant matrix and are contained within the mass of the nutritional components (e.g., carbohydrates).

## Data Availability

The original contributions presented in this study are included in the article. Further inquiries can be directed to the corresponding author.
